# Surgery

**Published:** 1895-03

**Authors:** 


### SURGERY.

EDITED BY FLOYD W. McRAE, M.D.

### THE SURGERY OF THE SPINAL CORD AND ITS APPENDAGES.

William Thorburn reviewed the present position and future prospects of the surgery of the spinal cord and its appendages in two lectures recently delivered before the Royal College of Surgeons of England (*British Medical Journal*, 1894, Nos. 1747 and 1748). The term "laminectomy," it is pointed out, is of quite recent creation, but is adopted throughout as more suitable than the older terms, "trephining the spine," "vertebral resection," "rachiotomy," etc. "Laminectomy" is preferable, but has not been generally accepted. The author says, "The dangers of the operation are not great, especially in view of the conditions which it is intended to relieve."

*Mortality.* Of seventy cases of tuberculous affections for which laminectomy was performed by distinguished surgeons, in twelve instances death is said to have been due to or hastened by the operation—a mortality of 17.1 per cent. The death was in most cases attributed to shock. Sepsis has been practically absent, and hemorrhage has rarely been serious.

*Penetrating wounds of the Cord.* Of forty published cases of stab-wounds, in thirty-eight of which the result is stated, but fifteen died—in nine instances from septic infection. In twenty-one non-fatal cases of wounds of the cord, all under the most favorable circumstances, but three fully recovered; sixteen had persistent paralysis or anesthesia, or both, and two more were said to be improving when lost sight of. This justifies the general conclusion that in man we cannot hope for anatomical recovery after a lesion of the spinal cord. The case is different with the nerve-roots, however, after they have left the cord. In the event of injury these may be sutured with every prospect of restoration of function.

*Fractures and dislocations.* In compound fractures (usually gun-shot wounds) there can be no doubt of the advisability of removing all splinters and foreign bodies. Of the fractures of the spine, those of the laminæ are the most important. From the analysis of ten published cases, the author arrives at the following conclusions: 1. The cause of this injury has generally been stated to be direct violence to the spine, but in four of my ten cases there was certainly no direct violence, and in only three is such distinctly described. Further, the frequency of this injury in conjunction with a fracture of the bodies indicates a similarity in the mechanism of their production. 2. The symptoms of fracture of the laminæ are by no means definite, and the only case in which we can be satisfied that the diagnosis was made before death, owed its detection to the distinct lateral mobility of a spinous process. 3. The diagnosis is therefore difficult, and probably we can rely only on the following points: *a.* The cause, if there be a clear history of direct violence. *b.* The normal contour of the spine and the presence of lateral mobility of one or more of the spinous processes in conjunction with an obvious lesion of the spinal cord.

In these cases laminectomy is clearly indicated, if there be any symptoms of pressure on the cord.

Fractures of the bodies of the vertebræ and dislocations are considered together. The displacement in dislocation is said to be forward and downward; any other form may be regarded as a pathological curiosity.

*Nature of injury to the Cord.* Such being the main varieties of injury to the spine, we may now ask ourselves what are the various methods in which the cord itself suffers, and we find these to be as follows:

1. By far most commonly there is approximation of the laminæ of the vertebræ above to the body of that below, causing crushing of the cord. Such crushing may be associated, on the one hand, with permanent pressure, as in true dislocation and in fractures which do not recoil; or, on the other, with temporary compression, as in diastases and in fractures which do recoil.

2. A fragment of bone may be driven back upon the spinal cord, but such a condition is exceedingly rare.

3. Equally rare, if not more so, is a condition presented in these two specimens, in which an intervertebral disk is, as it were, squeezed out from between the adjacent bones, so as to form a projecting shelf which compresses the theca.

4. And lastly, the medullary symptoms may be mainly due to the pressure of hemorrhage.

The diagnosis of these different lesions is as yet impracticable. The condition known as gravitating hemorrhage is easily recognized. It is met with in cases in which some of the spinal blood-vessels are injured, and as the blood escapes it gravitates to the lowest portion of the spinal canal, causing paralysis by pressure. The paralysis rapidly extends upward as the collection of blood rises in the canal. The author would be inclined to perform laminectomy at the seat of injury and attempt to arrest all visible bleeding points, and give exit to the blood that could not be arrested. This procedure may be combined with paracentesis of the meninges in the lumbar region after Quincke's method, or, if this was not satisfactory, a secondary laminectomy at the lower part of the spine.

Practically there are three varieties of injury of the cord which call for treatment, namely: Permanent pressure upon the cord, temporary compression of the cord, and hemorrhage. The latter is rare and difficult to diagnosticate. In the first condition it is possible to restore the normal lumen of the canal, but it is doubtful if any benefit will result, as the injured cord is not capable of regeneration. In the cases of temporary pressure, which constitute the majority, nothing could, of course, be obtained by operation. The author expresses the opinion that we are not yet able to treat with success the common injuries of the spinal cord. The author summarizes his conclusions as follows: In compound fractures, operate. In fractures of the spinous processes and laminæ, with injury to the cord, we also operate. In simple fractures and dislocations of the bodies of the vertebræ, if there is a reasonable probability that the injury is due to hemorrhage, operation is advisable; but in all other cases of this nature we cannot hope to do good save where the injury is below the level of the first lumbar vertebræ. In such cases, however, laminectomy is an eminently valuable surgical procedure. —*Am. Jour. Med. Science.*

